# The Functional *−765G→C* Polymorphism of the *COX-2* Gene May Reduce the Risk of Developing Crohn's Disease

**DOI:** 10.1371/journal.pone.0015011

**Published:** 2010-11-24

**Authors:** Hilbert S. de Vries, Rene H. M. te Morsche, Martijn G. H. van Oijen, Iris D. Nagtegaal, Wilbert H. M. Peters, Dirk J. de Jong

**Affiliations:** 1 Department of Gastroenterology and Hepatology, Radboud University Nijmegen Medical Center, Nijmegen, The Netherlands; 2 Department of Pathology, Radboud University Nijmegen Medical Center, Nijmegen, The Netherlands; 3 Department of Gastroenterology and Hepatology, University Medical Center Utrecht, Utrecht, The Netherlands; Charité-University Medicine Berlin, Germany

## Abstract

**Background:**

Cyclooxygenase-2 (COX-2) is a key enzyme involved in the conversion of arachidonic acid into prostaglandins. COX-2 is mainly induced at sites of inflammation in response to proinflammatory cytokines such as interleukin-1α/β, interferon-γ and tumor necrosis factor-α produced by inflammatory cells.

**Aim:**

The aim of this study was to investigate the possible modulating effect of the functional *COX-2* polymorphisms *−1195 A→G* and −*765G→C* on the risk for development of inflammatory bowel disease (IBD) in a Dutch population.

**Methods:**

Genomic DNA of 525 patients with Crohn's disease (CD), 211 patients with ulcerative colitis (UC) and 973 healthy controls was genotyped for the *−1195 A→G* (rs689466) and −*765G→C* (rs20417) polymorphisms. Distribution of genotypes in patients and controls were compared and genotype-phenotype interactions were investigated.

**Results:**

The genotype distribution of the *−1195A→G* polymorphism was not different between the patients with CD or UC and the control group. The −*765GG* genotype was more prevalent in CD patients compared to controls with an OR of 1.33 (95%CI 1.04–1.69, p<0.05). The *−765GC* and *−765CC* genotype carriers showed a tendency to be less frequent in patients with CD compared to controls, with ORs of 0.78 (95%CI: 0.61–1.00) and 0.49 (95%CI 0.22–1.08), respectively. Combining homozygous and heterozygous patients with the *−765C* allele showed a reduced risk for developing CD, with an OR of 0.75 (95%CI: 0.59–0.96). In the context of this, the G_−1195_G_−765_/A_−1195_C_−765_ diplotype was significantly less common in patients with CD compared to controls, with an OR of 0.62 (95%CI: 0.39–0.98). For UC however, such an effect was not observed. No correlation was found between COX-2 diplotypes and clinical characteristics of IBD.

**Conclusions:**

The −*765G→C* polymorphism was associated with a reduced risk for developing Crohn's disease in a Dutch population.

## Introduction

Inflammatory bowel disease (IBD) is an idiopathic, chronic, relapsing auto inflammatory disorder of the gastro-intestinal tract. The two major types of IBD are Crohn's disease (CD) and ulcerative colitis (UC). Genetic, immunological and environmental factors are thought to play a role in the pathogenesis of IBD [1]. A dysregulated immune response against the intestinal microbiota in genetic susceptible individuals has been heavily implicated in the pathogenesis of inflammatory bowel disease [2]. Therefore, genes involved in inflammatory responses are under investigation to look for variants predisposing to IBD.

Cyclooxygenase (COX) is a modifier gene and key enzyme in the conversion of free arachidonic acid into prostaglandins and is involved in the regulation of inflammatory processes through its products, mainly prostaglandin E_2_ (PGE_2_) [3]. The COX family consists of two main isozymes: COX-1 and COX-2. COX-1 is constitutively expressed in most cell types, including the mucosal compartment of the gastrointestinal tract, and is important for maintaining mucosal integrity, mucosal defence and regulation of the mucosal blood flow [4], [5]. Being very low expressed in the normal gut mucosa, COX-2 expression can be induced by mitogenic and proinflammatory stimuli [5], [6].

The relevance of COX-2 in the pathogenesis of IBD has been demonstrated; increased expression of COX-2 has been observed in colonic epithelial cells, the myenteric plexus and in the medial layer of arteries from patients with active IBD [7]–[9]. In addition, a relationship between endoscopic activity of IBD and mucosal COX-2 mRNA levels was noticed [10]. Although COX-2 is involved in the regulation of inflammatory processes, it also seems to play a physiological role in the defence of the gastric mucosa, as well as in the maintenance of gastric mucosal integrity when other defence mechanisms are impaired or COX-1 activity is latent [3], [5]. Moreover, COX-2 seems to be a major contributor to the processes that lead to resolution of inflammation [11]. In line with this, the use of non-steroidal anti-inflammatory drugs (NSAIDs) in patients with IBD, may be associated with exacerbation of the underlying IBD and gastrointestinal-related complications [12]–[14]. Overall, these findings suggest that COX-2 has a dual role by both initiation as well as resolution of inflammation.

Functional polymorphisms in the *COX-2* promoter, being −*765G→C* (rs20417) and *−1195A→G* (rs689466), may alter the enzyme function of COX-2 by differential regulation of COX-2 expression [15]. Recently, a study by Østergaard et al. reported an association of the *−765G→C* polymorphism with IBD in a Danish population [16]. Another study from a previous relatively small sample size study performed in the Netherlands however, showed no association between these two polymorphisms and IBD [17]. We therefore investigated the *COX-2 −1195 A→G* and −*765G→C* polymorphisms in relation to the development and clinical severity of IBD in a phenotypically well characterized and relatively large IBD cohort of Dutch origin and hypothesized that carriers of the *−1195 A→G* and/or −*765G→C* polymorphisms might be at risk for developing IBD.

## Materials and Methods

### Patients and controls

This case-control study included 736 patients with inflammatory bowel disease (39% men, mean age 45.0±13.9 years), being 525 patients with Crohn's disease (35% men, mean age 44.5±13.9) and 211 patients with ulcerative colitis (48% men, mean age 46.1±14.0) and 973 disease-free controls (43% men, mean age 47.2±16.6 years). All patients were of Dutch origin and were recruited from the outpatient clinic of the Radboud University Nijmegen Medical Center, the Netherlands. Controls were recruited from the Nijmegen area by advertisement in local papers. The clinical characteristics of the patients are summarized in Tables 1 and 2. Diagnosis of inflammatory bowel disease was based on accepted clinical, endoscopic, radiological and histological findings [18]. Clinical data of the patients were retrieved by retrospective collection from patients' clinical charts. Phenotypes of the patients were described according to age of onset, necessity of surgery, family history of IBD, the occurrence of extra-intestinal manifestations and maximum extent of disease according to the Vienna [19] and Montreal [20] classifications for Crohn's disease and ulcerative colitis respectively.

**Table 1:** Clinical characteristics of patients with Crohn's disease (n = 525).

Characteristics (%)	
Age at diagnosis	27.1±10.7
Family history of IBD*	75/272 (27.6)
Disease localization	
Ileal	187 (35.6)
Colonic	127 (24.2)
Ileocolonic	211 (40.2)
Isolated upper disease+	36 (6.9)
Disease behaviour CD	
Non stricturing, non penetrating	176 (33.5)
Stricturing	89 (17.0)
Penetrating	260 (49.5)
Extra-intestinal disease*	161/491 (32.8)
Peri-anal disease*	180/509 (35.4)
Surgery	320 (61.0)

+ Patients could be classified as having disease localisation in the upper gastrointestinal tract next to ileal, colonic or ileocolonic localisation.

*Note that data of patients are missing.

**Table 2:** Clinical characteristics of patients with Ulcerative Colitis (n = 211).

Characteristics (%)	
Age at diagnosis	32.9±12.7
Disease localization*	
Proctitis	13/203 (6.4)
Left sided	70/203 (34.5)
Extended/pancolitis	120/203 (59.1)
Surgery	59 (28.0)

*Note that data of 8 patients are missing.

Information on development of dysplasia and colorectal cancer (CRC) in our patient cohort was retrieved using PALGA, the nationwide network and registry of histopathology and cytopathology in the Netherlands [21].

The ethical committee of region Nijmegen and Arnhem reviewed and approved the protocol under number CWOM-nr 9804-0100. Verbal informed consent was obtained from each patient before study participation in agreement with the approval and all samples were anonymized. Since research data were collected anonymously, at least verbal informed consent was needed according to national regulations. Therefore, no written informed consent procedure was introduced at time of data collection.

### Genotyping

Whole blood from patients and healthy controls was obtained by venapuncture in sterile vacutainer tubes, anti-coagulated with EDTA and stored at −20°C until use. DNA from patients and controls was isolated from whole blood using the Pure Gene DNA isolation kit, according to the instructions of the manufacturer (Gentra Systems, Minneapolis, MN) and stored at 4°C. Genotypes of the *COX-2* −*1195A→G* polymorphism was determined by polymerase chain reaction (PCR)-based restriction fragment length polymorphism assays, as described by Zhang *et al* [15]. The *COX-2* −*765G→C* polymorphism was determined by a dual-color discrimination assay using the iCycler iQ Multicolour Real-Time Detection System (Bio-Rad Laboratories, Hercules, CA), as described by Peters *et al* [22].

### Statistical analysis

Baseline and clinical characteristics were analysed with standard descriptive statistics. The observed genotype frequencies were tested for deviation from the Hardy-Weinberg equilibrium. Estimates of linkage disequilibrium (LD) between SNPs were determined by calculating pair-wise D′ and r^2^ statistics in unrelated individuals, using Haploview. Differences in *−1195A→G* and *−765G→C* genotype distributions between the patient and control groups were determined by Chi-square analysis. Odds ratios (ORs) with 95% confidence interval (95% CI) were calculated for genotypes associated with predicted normal versus predicted altered enzyme activities (variant genotypes) between IBD patients and controls. These analyses were also applied for testing of either UC or CD with the control group. Based on the two polymorphisms investigated, a diplotype analysis was performed. Diplotypes were compared with regard to phenotypical characteristics and comparisons were given as ORs with 95% CI. Additionally, we investigated in patients with IBD whether the *−1195A→G* and *−765G→C* polymorphisms were associated with development of mucosal dysplasia or colon cancer. Data analysis was performed using SPSS software (Version 16.0, SPSS, Chicago, IL, USA). A p-value of <0.05 was used as a criterion for statistical significance.

## Results

In this study 736 patients with inflammatory bowel disease, 525 patients with Crohn's disease and 211 patients with ulcerative colitis as well as 973 healthy controls were included. No statistical significant differences were observed between patients with IBD and controls regarding age and gender. However when the CD or UC patient groups were compared to controls separately, significant more females were present in the group with Crohn's disease (p<0.01).

Distribution of the *−1195* and *−765 COX-2* genotypes in both patient and control groups fitted the Hardy Weinberg equilibrium; for the *−1195* genotypes, p-values of p = 0.14, p = 0.17 and p = 0.99, for the patients with Crohn's disease, ulcerative colitis and controls were found; whereas corresponding p-values for the *−765* genotypes were p = 0.64, p = 0.26 and p = 0.87, respectively. As been reported before by others [15], [17], [23], both SNPs were found to be in strong linkage disequilibrium (*D′* = 1, *r^2^* = 0.05).

### Genotype distribution and association with inflammatory bowel disease

The distribution of the *−1195* and *−765 COX-2* genotypes as found in patients with IBD and controls is given in Table 3. The *−1195* genotype distribution was not different between the patients with Crohn's disease, ulcerative colitis, or all IBD patients taken together in comparison with the control group. However, the *−765* genotype distribution showed a tendency towards a significant difference between patients with Crohn's disease and controls, with the *−765GC* and *−765CC* genotypes being less prevalent in patients, with ORs of 0.78 (95%CI 0.61–1.00, p<0.05) and 0.49 (95%CI 0.22–1.08) respectively and the *−765GG* genotype being more prevalent in patients (OR 1.33, 95%CI 1.04–1.69, p<0.05). No differences were found between patients with ulcerative colitis and controls. Combining homozygous (*−765CC*) and heterozygous (*−765GC*) patients bearing the *−765C* allele, showed a reduced risk for developing Crohn's disease in this group (OR = 0.75, 95%CI 0.59–0.96, p<0.05).

**Table 3:** Distribution of the *COX-2 −1195* and *−765* genotypes and corresponding ORs in patients with IBD, CD or UC versus controls.

Genotype *COX-2*	All patients with IBD (n = 736)			Patients with Crohn's disease (n = 525)			Patients with Ulcerative Colitis (n = 211)+			Controls
	Number (%)	OR (95% CI)	p-value	Number (%)	OR (95% CI)	p-value	Number (%)	OR (95% CI)	p-value	n = 973 (%)
*−1195AA*	476 (64.7)	Reference	-	339 (64.6)	Reference	-	137 (64.9)	Reference	-	618 (63.5)
*−1195GA*	221 (30.0)	0.91 (0.74–1.12)	0.38	159 (30.3)	0.92 (0.73–1.16)	0.48	62 (29.4)	0.89 (0.64–1.23)	0.48	315 (32.4)
*−1195GG*	39 (5.3)	1.27 (0.80–2.00)	0.31	27 (5.1)	1.23 (0.74–2.04)	0.42	12 (5.7)	1.35 (0.69–2.65)	0.38	40 (4.1)
*−765GG*	535 (73.2)	Reference	-	394 (75.0)	Reference	-	141 (68.4)	Reference	-	675 (69.4)
*−765GC*	179 (24.5)	0.84 (0.67–1.04)	0.11	123 (23.4)	0.78 (0.61–1.00)	0.05	56 (27.2)	0.99 (0.71–1.40)	0.97	270 (27.7)
*−765CC*	17 (2.3)	0.77 (0.42–1.41)	0.39	8 (1.5)	0.49 (0.22–1.08)	0.07	9 (4.4)	1.53 (0.71–3.33)	0.27	28 (2.9)

+ In the ulcerative colitis group, there are some missing data (n = 5) due to unsuccessful PCR for the *−765 G→C* polymorphism.

OR = Odds ratio; CI = confidence interval.

The effects of the two COX-2 polymorphisms were then studied in the context of diplotypes. Six diplotypes were identified, with the A_−1195_G_−765_/A_−1195_G_−765_ diplotype being the most prevalent in both patients and controls (Table 4). The G_−1195_G_−765_/A_−1195_C_−765_ diplotype was significantly less frequent in patients with Crohn's disease compared to controls with an OR of 0.62 (95%CI: 0.39–0.98, p<0.05).

**Table 4:** *COX-2* diplotype distribution and corresponding ORs in patients with IBD, CD or UC versus controls.

				Patients with IBD						
Diplotype *COX-2*	All patients			Crohn's disease			Ulcerative colitis			Controls
	n = 731 (%)	OR (95% CI)	p-value	n = 525 (%)	OR (95%CI)	p-value	n = 206 (%)	OR (95%CI)	p-value	n = 973 (%)
*A_−1195_G_−765_/A_−1195_G_−765_*	322 (43.8)	Reference	-	237 (45.1)	Reference	-	85 (40.3)	Reference	-	395 (40.6)
*G_−1195_G_−765_/A_−1195_G_−765_*	174 (23.6)	0.90 (0.70–1.15)	0.38	130 (24.8)	0.91 (0.70–1.19)	0.49	44 (20.9)	0.86 (0.58–1.28)	0.45	238 (24.5)
*A_−1195_G_−765_/A_−1195_C_−765_*	133 (18.1)	0.84 (0.65–1.10)	0.20	94 (17.9)	0.81 (0.60–1.08)	0.15	39 (18.5)	0.93 (0.62–1.42)	0.75	194 (19.9)
*G_−1195_G_−765_/A_−1195_C_−765_*	46 (6.5)	0.72 (0.49–1.07)	0.11	29 (5.5)	0.62 (0.39–0.98)	0.04	17 (8.1)	1.01 (0.57–1.80)	0.97	78 (8.0)
*G_−1195_G_−765_/G_−1195_G_−765_*	39 (5.3)	1.20 (0.75–1.90)	0.45	27 (5.1)	1.13 (0.67–1.88)	0.65	12 (5.7)	1.39 (0.70–2.77)	0.34	40 (4.1)
*A_−1195_C_−765_/A_−1195_C_−765_*	17 (2.3)	0.75 (0.40–1.39)	0.35	8 (1.5)	0.48 (0.21–1.06)	0.06	9 (4.3)	1.49 (0.68–3.28)	0.32	28 (2.9)

OR = Odds ratio; CI = confidence interval.

### Correlation of the COX-2 diplotypes with clinical characteristics of IBD patients

Additionally, clinical characteristics of patients with Crohn's disease and ulcerative colitis were studied in the context of diplotypes in which the most common A_−1195_G_−765_/A_−1195_G_−765_ diplotype served as reference. No significant association between the *COX-2* diplotypes and clinical characteristics of either Crohn's disease or ulcerative colitis was found (Tables 5 and 6). When data were corrected for age and gender, no significant changes in data were observed.

**Table 5:** Diplotype-phenotype correlations in patients with Crohn's disease.

	*AG/AG* *	*GG/AG*	Odds ratio	p-value	*AG/AC*	Odds ratio	p-value	*GG/AC*	Odds ratio	p-value	*GG/GG*	Odds ratio	p-value	*AC/AC*	Odds ratio	p-value
			(95%CI)			(95%CI)			(95%CI)			(95%CI)			(95%CI)	
**Disease localization (n = 525)**																
Ileal	83	51	Reference	-	34	Reference	-	8	Reference	-	8	Reference	-	3	Reference	-
Colonic	63	26	0.67 (0.38–1.19)	0.17	23	0.89 (0.48–1.66)	0.72	8	1.32 (0.47–3.70)	0.60	6	0.99 (0.33–2.99)	0.98	1	0.44 (0.05–4.32)	0.47
Ileocolonic	91	53	0.95 (0.58–1.54)	0.83	37	0.99 (0.57–1.73)	0.98	13	1.48 (0.59–3.76)	0.41	13	1.48 (0.59–3.76)	0.41	4	1.22 (0.26–5.60)	0.80
Isolated upper disease+	18	7	0.63 (0.25–1.62)	0.34	8	1.09 (0.43–2.73)	0.86	2	1.15 (0.23–5.89)	0.86	1	0.58 (0.07–4.90)	0.61	0	-	0.42
**Disease behavior (n = 525)**																
Non stricturing/	75	44	Reference	-	37	Reference	-	10	Reference	-	8	Reference	-	2	Reference	-
penetrating																
Stricturing	43	18	0.71 (0.37–1.39)	0.32	14	0.66 (0.32–1.36)	0.26	8	1.40 (0.51–3.80)	0.51	4	0.87 (0.25–3.07)	0.83	2	1.74 (0.24–12.83)	0.58
Penetrating	119	68	0.97 (0.61–1.57)	0.91	43	0.73 (0.43–1.24)	0.25	11	0.69 (0.28–1.71)	0.43	15	1.18 (0.48–2.92)	0.72	4	1.26 (0.23–7.05)	0.79
**Surgery**	146	87	1.26 (0.81–1.98)	0.31	50	0.71 (0.44–1.15)	0.16	16	0.77 (0.35–1.67)	0.50	17	1.06 (0.47–2.42)	0.89	4	0.62 (0.15–2.55)	0.51
**Extra-intestinal (n = 491)**	77	32	0.69 (0.43–1.13)	0.14	37	1.39 (0.84–2.30)	0.21	8	0.80 (0.34–1.92)	0.62	6	0.64 (0.24–1.67)	0.36	1	0.27 (0.03–2.26)	0.20
**Family history of IBD (n = 272)**	36	18	0.82 (0.43–1.59)	0.57	14	1.08 (0.52–2.26)	0.84	2	0.62 (0.13–3.05)	0.55	3	0.67 (0.18–2.56)	0.56	2	1.65 (0.26–10.28)	0.59
**Dysplasia**	7	4	1.04 (0.30–3.63)	0.95	1	0.35 (0.04–2.91)	0.31	1	1.17 (0.14–9.89)	0.88	2	2.63 (0.52–13.35)	0.23	0	-	0.62
**Colon cancer**	4	3	0.73 (0.16–3.30)	0.68	0	-	0.21	0	-	0.48	0	-	0.50	0	-	0.71

Diplotype-phenotype correlations in patients with Crohn's disease in which the AG/AG diplotype served as reference.

+ Patients could be classified as having disease localisation in the upper gastrointestinal tract next to ileal, colonic or ileocolonic localisation.

* For full notation see Table 4.

**Table 6:** Diplotype-phenotype correlations in patients with ulcerative colitis.

	*AG/AG* #	*GG/AG*	Odds ratio (95%CI)	p-value	*AG/AC*	Odds ratio (95%CI)	p-value	*GG/AC*	Odds ratio (95%CI)	p-value	*GG/GG*	Odds ratio (95%CI)	p-value	*AC/AC*	Odds ratio (95%CI)	p-value
**Disease localization (n = 198)**																
Proctitis	6	2	Reference	-	2	Reference	-	2	Reference	-	1	Reference	-	0	Reference	-
Left sided	27	13	1.44 (0.26–8.16)	0.676	15	1.67 (0.30–9.31)	0.558	7	0.78 (0.13–4.72)	0.784	3	0.67 (0.06–7.57)	0.742	4	-	0.351
Pancolitis	46	28	1.83 (0.35–9.68)	0.474	21	1.37 (0.26–7.36)	0.713	8	0.52 (0.09–3.06)	0.465	8	1.04 (0.11–9.86)	0.970	5	-	0.422
**Surgery (n = 206)**	24	11	0.85 (0.37–1.94)	0.695	11	1.00 (0.43–2.32)	0.997	8	2.26 (0.78–6.54)	0.127	2	0.51 (0.10–2.49)	0.397	2	0.73 (0.14–3.75)	0.701
**Dysplasia (n = 211)**	5	3	1.17 (0.27–5.14)	0.835	2	0.87 (0.16–4.67)	0.866	0	-	0.305	1	1.46 (0.16–13.63)	0.741	2	4.57 (0.75–28.01)	0.076

Diplotype-phenotype correlations in patients with ulcerative colitis in which AG/AG served as reference.

# For full notation see Table 4.

### COX-2 polymorphisms and the risk for developing dysplasia and colon cancer in patients with inflammatory bowel disease

The PALGA search regarding dysplasia and colon cancer in our IBD cohort demonstrated that 29 patients (15 patients with CD and 14 patients with UC) developed mucosal dysplasia, which is regarded as a pre-malignant phase of CRC. Furthermore, in the CD cohort 7 patients with CRC were identified; 4 having the A_−1195_G_−765_/A_−1195_G_−765_ diplotype and 3 having the G_−1195_G_−765_/A_−1195_G_−765_ diplotype. In the UC cohort, no patients were identified who developed CRC. When tested, no association was found between the COX-2 diplotypes and the development of colonic dysplasia or cancer (Tables 5 and 6).

## Discussion

This study was performed to determine the possible modulating effect of the *COX-2* −*1195 A→G* and −*765G→C* polymorphisms on the risk of developing inflammatory bowel disease. Carriers of the *−765C* allele showed a reduced risk for developing CD. This result suggests that the −*765G→C* change induces an altered enzyme expression and enzyme activity with potential anti-inflammatory consequences.

Studies regarding the functional consequences of the −*765G→C* polymorphism in the *COX-2* promoter are conflicting. Therefore, the (physiological) consequences of our findings are difficult to interpret. First of all, the *−765C*-containing COX-2 promoter was reported to drive lower reporter gene expression in vitro compared to the *−765G*-containing counterpart [15], [24]. Furthermore, serum prostaglandin E_2_ (PGE_2_) concentrations of renal transplant recipients patients with the GG genotype were significantly higher than PGE_2_ concentrations from patients with the C allele [25]. Subsequent work from Zhang and coworkers showed that the −765G→C polymorphism creates a binding site for nucleophosmin (NPM) and phosphorylated nucleophosmin (p-NPM), which acts as an inhibitor of COX-2 transcription [26]. The −1195 A→G polymorphism creates a c-MYB binding site, which can activate COX-2 expression, and displays a higher promoter activity [15].

In normal colorectal mucosa COX-2 expression is enhanced in patients with IBD when compared to subjects with normal colonoscopy [27]. Taken together in light of our results, this would imply that low levels of COX-2 are associated with an reduced risk for developing CD. In vitro however, when cells were treated with smoking condensate, the *−765C*-containing promoter exerted a significantly higher reporter gene expression compared to the *−765G*-containing counterpart [26]. Besides this, Szczeklik and co-workers reported an increased production of prostaglandin E_2_ and D_2_ (PGE_2_ and PGD_2_) by monocytes obtained from female patients with asthma who were homozygous for the *−765C* variant of the COX-2 gene [28], [29]. In the context of IBD, PGE_2_ appears to play a dual role. In IBD, PGE_2_ production is increased [30] and in an experimental model of IBD high levels of PGE_2_ exacerbate inflammation [31]. On the other hand, PGE_2_ signaling is required for suppressing colitis symptoms and protecting mucosal damage by maintaining the integrity of the epithelial intestinal wall, presumably through the enhancement of epithelial survival and regeneration [32]. Furthermore, PGE_2_ has been recently identified to promote naive T cell differentiation to IL-17 – producing T helper (Th17) cells, a subset of T helper cells which have been implicated as potent effector cells in IBD [33].

Several limitations of our study should be noticed. First of all we were not able to retrieve the smoking status of our patients and controls, as Zhao et al. [26] demonstrated an effect of smoking on the expression of the *−765G→C* polymorphism. Secondly, the effect of the *COX-2* −*1195 A→G* and −*765G→C* polymorphisms on colonic mucosal COX-2 expression and/or PGE_2_ production in patients with IBD is unknown. However regardless of these data, the functional consequences of PGE_2_ in IBD still remains conflicting as pointed out above.

The results of our study are in conflict with a Danish case control study by Østergaard et al. who identified that carriers of the homozygous *−765CC* variant had a relatively high risk for developing CD as well as UC, with an OR of 2.78 (95%CI = 1.33–5.88, p = 0.006) and 2.63 (95%CI = 1.35–5.26, p = 0.005) respectively [16]. The *−765CC* variant however is very rare in our population of IBD patients (n = 17, 2.3%) and controls (n = 28, 2.9%) as is the case in another Dutch study by Cox et al. in which (2.4%) of the patients and (2.4%) of the controls had this variant [17]. In the study of Cox et al., no significant association between the *−1195 A→G* and −*765G→C* polymorphisms and IBD was found, although the number of patients with IBD involved (n = 291) was rather small. However, a recent subsequent study from the Danish group of Østergaard and co-workers extended the original data with data from Scottish IBD patients and showed no association any more with the −*765G→C* polymorphism and development of IBD [34]. The differences between our results and the Danish and Scottish findings could be attributed to the fact that the genetical contribution to the etiology of IBD in the northern part of Europe differs from central Europe. Mutations in the three common CD-associated variants of CARD15, R702W, G908R and 1007fsinsC, are relatively rare in Northern countries including Denmark and Scotland, while the mutation frequencies are relatively high in Central Europe [35].

As stated before, patients with IBD show increased expression of COX-2 in the gastrointestinal tract [7], [8], [10], [27]. This increased expression of COX-2 has also been observed in gastrointestinal adenocarcinomas and in UC-associated neoplasia [36], [37]. Additionally, the *COX-2 −1195 A→G* and −*765G→C* polymorphisms were demonstrated to influence the expression of COX-2 and confer a risk for developing (adeno)carcinomas in the gastrointestinal tract [15], [38], [39]. Chronic intestinal inflammation-associated colorectal carcinogenesis is thought to occur via a stepwise progression beginning with epithelial hyperplasia, leading to various grades of dysplasia, adenoma, and then to adenocarcinoma [40]. We investigated whether or not an association could be found between the *COX-2* polymorphisms and dysplasia or CRC in patients with IBD. Due to the restricted number of patients who developed dysplasia or CRC, no differences could be observed.

In conclusion, subjects with the *−765C* allele showed a reduced risk for developing CD. No correlation could be found between the *COX-2* diplotypes and clinical characteristics of IBD patients and the development of colonic dysplasia or cancer. Further studies are required to confirm the association we found and efforts should be made to unravel the role of COX-2 and its derived prostaglandins in the pathogenesis of IBD.
